# Dielectric relaxation and optical modulation in PVC/TPU-BaSnO_3_/Sn_2_O_3_ nanocomposites for enhanced energy storage and optoelectronic performance

**DOI:** 10.1039/d5ra06775h

**Published:** 2026-02-04

**Authors:** Nawal K. Almaymoni, Eman A. Mwafy, Ameenah N. Al-Ahmadi, Doaa Abdelhameed, Ayman A. O. Younes, Haitham alrajhi, Sherif S. Nafee, Ayman M. Mostafa

**Affiliations:** a Department of Physics, College of Science, Princess Nourah Bint Abdulrahman University P.O. Box 84428 Riyadh 11671 Saudi Arabia; b Physical Chemistry Department, Advanced Materials Technology and Mineral Resources Research Institute, National Research Centre 33 El Bohouth St., Dokki Giza 12622 Egypt; c Department of Physics, College of Sciences, Umm AL-Qura University 24382 Makkah Saudi Arabia; d Department of Physics, College of Science and Humanities, Prince Sattam Bin Abdulaziz University P.O. Box 173 Al-Kharj 11942 Saudi Arabia; e Department of Chemistry, College of Science, University of Bisha Bisha 61922 Saudi Arabia; f Department of Physics, College of Sciences, Imam Mohammad Ibn Saud Islamic University (IMSIU) P.O. Box 90950 Riyadh 11623 Saudi Arabia; g Physics Department, Faculty of Science, King Abdulaziz University 21589 Jeddah Saudi Arabia; h Department of Physics, College of Science, Qassim University P.O. Box 6644 Buraydah Almolaydah 51452 Saudi Arabia a.mostafa@qu.edu.sa; i Spectroscopy Department, Physics Research Institute, National Research Centre 33 El Bohouth st., P.O. 12622, Dokki Giza Egypt

## Abstract

This work involves the fabrication and comprehensive characterization of flexible polyvinyl chloride (PVC)/thermoplastic polyurethane (TPU)/BaSnO_3_/Sn_2_O_3_ (BSO/SO) nanocomposites, with a focus on optimizing their structural, dielectric relaxation, and optical properties for energy storage and optoelectronic applications, and they were synthesized by co-precipitation and drop-casting procedures. The distinctive systems were examined using XRD, FTIR, HRTEM, and FESEM techniques, including the structural integrity of blends and nanocomposites. A BaSnO_3_/Sn_2_O_3_ (BSO/SO) nanocomposite has been effectively integrated into the PVC/TPU blend, as demonstrated by microstructural characterization using XRD, EDX, and ATR-FTIR tools. Supplementation with nanoparticles elevated the crystallinity, improved interfacial contact, and optimized the filler distribution. The incorporation of BSO-SO nanofiller notably modifies the dielectric relaxation characteristics of PVC/TPU blends *via* mechanisms that include interfacial polarization, limited polymer mobility, and improved charge carrier dynamics, demonstrating a dielectric constant of 120, a twelvefold increase at higher concentrations of nanofiller at low frequencies. The amount of nanofiller in the PVC/TPU-BSO-SO composites greatly influences how they transmit and reflect light, improving their clarity, ability to block UV rays, and flexibility in terms of the optical properties. Pronounced reductions of the direct and indirect optical band gaps are seen. The changes in band gaps might be due to more disorder, changes in crystal structure, and the possible clumping of nanoparticles. The resulting nanocomposites combine flexibility with tunable dielectric and optical properties, positioning them as strong candidates for use in flexible energy storage, UV blocking, and optoelectronic applications.

## Abstract 2

This work involves the fabrication and comprehensive characterization of flexible polyvinyl chloride (PVC)/thermoplastic polyurethane (TPU)/BaSnO_3_/Sn_2_O_3_ (BSO/SO) nanocomposites for energy storage and optoelectronic applications.

## Graphical abstract

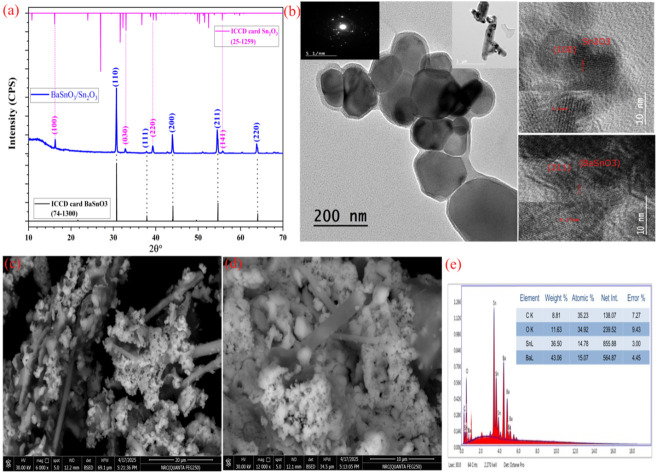

## 1 Introduction

In recent years, the advancement of flexible functional materials has garnered considerable interest owing to their prospective uses in wearable electronics, sensors, energy storage devices, optoelectronics, UV shielding, and other burgeoning technologies.^1,2^ Among these materials, polymer-based nanocomposites have emerged as particularly compelling options because of their distinctive combination of mechanical flexibility, processability, and tunable functional attributes.^3,4^ The advancement of sophisticated polymer blend nanocomposites with improved dielectric and optical characteristics has become a pivotal research domain for next-generation electronic and optoelectronic applications. A main focus of current studies is finding the best twin-screw extrusion settings, testing the durability over time, and scaling processes so they can be used in industry. Polyvinyl chloride (PVC), thermoplastic polyurethane (TPU), and recycled chemicals can be used in blends to make them more environmentally friendly. With these changes, PVC/TPU mixes are now recognized as flexible materials that can meet strict performance and environmental standards in a wide range of fields.^5–7^

PVC is the second most prevalent polymeric material owing to its chemical durability, affordability, and exceptional mechanical properties.^8^ Its characteristics render it appropriate for diverse commercial applications, such as synthetic leather, medical packaging, toys, shower curtains, kitchen flooring, piping, and window frames.^9^ PVC has poor flexibility and thermal stability, requiring the incorporation of plasticizers and stabilizers. Polymeric plasticizers provide an efficient resolution to this issue.^10^ The amalgamation of thermoplastic polyurethane (TPU) and PVC, both polar polymers exhibiting excellent compatibility, enhances product versatility as well as endurance at lower temperatures.^4,5,11^ This method diminishes the necessity for alternative low-molecular-weight plasticizers by leveraging the plasticizing properties of TPU. Despite the efficacy of small-molecule plasticizers, they may nevertheless migrate owing to inadequate adhesion and thermodynamic incompatibility.^5^

Strategically integrating ceramic nanoparticles, particularly perovskite materials with metal oxide systems, into polymer blend matrices has demonstrated efficacy for enhancing the dielectric and optical properties. Barium stannate (BaSnO_3_) is a promising material to add to other substances because of its special perovskite structure and excellent electrical properties. BaSnO_3_ has elevated dielectric constants and optical transparency, rendering it suitable for use as a transparent conducting oxide.^12,13^ When combined with BaSnO_3_, tin(ii) oxide (Sn_2_O_3_) nanoparticles offer additional advantages due to their distinctive optical and electrical characteristics. The intuitive incorporation of these two oxide systems into a flexible polymer blend matrix is an innovative approach for developing multifunctional nanocomposites with tailored properties.

Although numerous studies have investigated polymer nanocomposites that incorporate conventional ceramic fillers, including barium titanate, PbTiO_3_, and SnO_2_/SrSnO_3_, which demonstrate exceptional dielectric and ferroelectric properties,^14–20^ these systems frequently experience interfacial incompatibility, limited flexibility, and poor optical transparency, limiting their suitability for use in flexible or multifunctional optoelectronic devices.

In contrast, the current study introduces a hybrid PVC/TPU polymer matrix that is reinforced with BaSnO_3_/Sn_2_O_3_ nanostructures. This matrix combines the flexibility of TPU and the rigidity of PVC with the high dielectric and optical responsiveness of the perovskite-based infill. BaSnO_3_/Sn_2_O_3_ nanofillers are lead-free, chemically stable, and possess a tunable electronic structure, which enables enhanced interfacial polarization and reduced dielectric loss, in contrast to conventional BaTiO_3_- or Pb-based systems. Moreover, the dual-polymer framework enables enhanced nanoparticle dispersion and interphase coupling, resulting in synergistic modulation of the optical and dielectric properties. The study offers novel physical insights into the relationship between functional performance and microstructural evolution by utilizing a combination of XRD, HRTEM, FESEM, ATR-FTIR, dielectric, and UV-vis analyses. This research introduces a promising material design strategy for the development of high-performance, environmentally friendly, and flexible polymer nanocomposites that are specifically designed for advanced optoelectronic and energy-storage applications.

## 2 Experimental

### 2.1 Materials

Barium acetate (Ba(CH_3_COO)_2_) (Sigma-Aldrich 98%), sodium tin(iv) oxide trihydrate (Na_2_SnO_3_·3H_2_O), (Alfa Aesar 98%), polyvinyl chloride (PVC) with Mw ≈ 500 000 (Sigma-Aldrich), and thermoplastic polyurethane (TPU) were used. Tetrahydrofuran (THF) was utilized as a liquid medium for the synthesis of the PVC/TPU blend. All of the precursors are AR grade and used without any further purification.

#### 2.1.1 Preparation of the BaSnO_3_/Sn_2_O_3_ nanocomposite

Initially, 0.01 mol of barium acetate was dissolved in 30 mL of double-distilled water, and concurrently, 0.01 mol of sodium stannate trihydrate was dissolved in an additional 30 mL of double-distilled water as follows:

i Ba(CH_3_COO)_2_ (s) ⇄ Ba^2+^ + (aq) + 2CH_3_COO^−^(aq)

ii Na_2_SnO_3_·3H_2_O (s) → 2Na^+^ (aq) + SnO_3_^2−^ (aq) + 3H_2_O(i)

Solid barium acetate fully dissociates in water, yielding soluble barium ions (Ba^2+^) and acetate ions (CH_3_COO^−^). The process involves physical dissolution or ionic dissociation, rather than a chemical event characterized by bond creation or disintegration. When dissolved in double-distilled water, solid sodium stannate trihydrate dissociates into sodium ions (Na^+^) and stannate ions (SnO_3_^2−^), releasing three water molecules of crystallization into the solvent. The procedure involves ionic dissociation, yielding a transparent alkaline solution of sodium stannate.

Both solutions were continuously agitated with a magnetic stirrer for 4 hours, until they became transparent. The two solutions were combined, and the pH was adjusted to approximately 7–8 by the gradual addition of NaOH, resulting in the precipitation of a hydrated barium stannate precursor, followed by stirring for 5 h. A hydrated barium stannate precipitate occurs under mildly alkaline conditions (pH ≈ 7–8, controlled by the gradual addition of NaOH), followed by stirring for 5 h. Net ionic precipitation (formation of the hydrated precursor) occurs as follows:

iii Ba^2+^ + SnO_3_^2−^ (aq) + *x*H_2_O → BaSnO_3_·*x*H_2_O (s)

iv Ba(CH_3_COO)_2_ (aq) + Na_2_SnO_3_·3H_2_O (aq) → BaSnO_3_·*x*H_2_O (s) + 2CH_3_COONa (aq) + (3−*x*)H_2_O

The precipitate was filtered and air-dried at 90 °C for a duration of 12 h to remove much of the physically bound water. Thermal treatment at 450 °C for 4 h under an air atmosphere transforms the hydrated precursor into a crystalline oxide. The principal expected product is the perovskite BaSnO_3_/Sn_2_O_3_.

#### 2.1.2 Synthesis of polyvinyl chloride/thermoplastic blends incorporating the BaSnO_3_/Sn_2_O_3_ nanocomposite

At first, the desired quantity of PVC/TPU blend solution was generated by the traditional casting approach. Both powdered polymers, PVC and TPU, were solubilized in tetrahydrofuran (THF) and subsequently amalgamated and stirred for 4 hat 75 °C to attain a homogeneous solution.

Secondly, sonication was then used to incorporate BSO-SO nanoparticles into the PVC/TPU solution. These materials were denoted as PVC/TPU-0.04BSO-SO, PVC/TPU-0.07BSO-SO, PVC/TPU-0.1BSO-SO, and PVC/TPU-0.2BSO-SO. The weight percentages of BSO-SO solution were 0.04, 0.07, 0.1, and 0.2 wt%, respectively. For the purpose of producing nanocomposite films, the mixture produced was subsequently cast onto a Petri dish and subjected to a drying process at a temperature of 65 °C for 20 h.

### 2.2 Characterization

The structures of the materials were analysed *via* powder X-ray diffraction (PXRD) with an Empyrean Panalytical Instruments diffractometer from the Netherlands. CuK served as the X-ray radiation source. To identify the various functional groups present in the prepared nanocomposites, samples were obtained and analyzed using a Bruker Corporation apparatus from Germany, which includes a platinum diamond disc as the internal reflection element to obtain ATR-FTIR spectra in the range of 4000–400 cm^−1^. The material microstructures were analysed using a JEOL model JEM-2100 high-resolution transmission electron microscope (HRTEM) from Japan. The surface morphology was analyzed using field emission scanning electron microscopy (FESEM) with an energy dispersive X-ray (EDX) detector, namely the Quanta 250 FEG FEI model. The morphologies of the samples were studied by mounting them on a stainless-steel stub with double-sided carbon tape. To prevent charge accumulation, a gold (Au) coating was applied. The optical UV (transmission and reflection) spectra of the samples were recorded at ambient temperature using an optical spectrophotometer (Jasco model V-570) across a wavelength range of 190–2500 nm. For dielectric and electrical studies, the nanocomposite films were cut into circular disks (diameter: 10 mm) and coated on both sides with silver paste to form parallel-plate capacitor configurations. Broadband dielectric spectroscopy and AC conductivity measurements were performed using a Novocontrol high-resolution Alpha analyzer equipped with a Quatro temperature control system, allowing precise temperature-dependent measurements over a frequency range of 10^−1^–10^7^ Hz.

## 3 Results and discussion

### 3.1 X-ray diffraction, high-resolution transmission electron microscopy, and field emission scanning electron microscopy

**Fig. 1:**
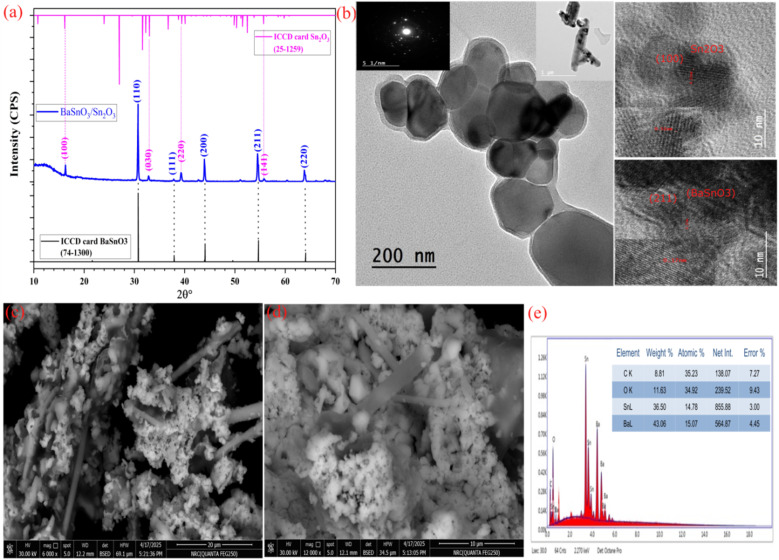
(a) The XRD pattern of the BaSnO_3_/Sn_2_O_3_ nanocomposite, (b) HRTEM images and the SAED pattern, and (c–e) FESEM images and the EDX spectrum of the nanocomposite.

The XRD pattern of the BaSnO_3_/Sn_2_O_3_ nanocomposite is depicted in Fig. 1(a). Crystalline behaviour is shown by the XRD spectrum of the material when it is heated to 450 °C in an air environment. A cubic phase is exhibited by the material, which corresponds to the perovskite BaSnO_3_ (JCPDS 74-1300), which crystallizes in the space group P23 (195).^12,13,21^ Some of the Bragg angles and indices that correlate to the observed peaks are as follows: 30.72° (110), 37.81° (111), 43.95° (200), 54.51° (211), and 63.96° (220). Due to the formation of the metal oxide semiconductor Sn_2_O_3_, one can notice smaller peaks that correlate to the Bragg angles and planes that are related to it. A triclinic phase corresponding to JCPDS 25-1259 is indicated by the peaks at angles of 16.21° (100), 32.89° (030), 39.30° (220), and 55.79° (141),^22,23^ and this phase is distinguished by the special space group P*(2). A great degree of purity was displayed by the samples that were produced, as there were no identifiable lines that were associated with contamination. The remarkable purity of the samples was considered as evidence of their successful synthesis.

Fig. 1(b) shows the use of HRTEM, lattice spacing analysis, and SAED to study the structure of the BaSnO_3_/Sn_2_O_3_ nanocomposite. HRTEM analysis verifies the crystallization pattern of the BaSnO_3_/Sn_2_O_3_ nanocrystals, which is characterized by elongated cubic and triclinic forms, as well as nanorods located in other positions (see Fig. 1(b)). The inset in Fig. 1(b) illustrates the selected area electron diffraction (SAED) pattern, which indicates the composite's polycrystalline structure. The observed lattice lines with a distance of 0.17 nm between them match the (211) plane of the cubic structure of BaSnO_3_, while those with a spacing of 0.51 nm match the (100) plane of the triclinic structure of Sn_2_O_3_, confirming the XRD data.

### 3.2 ATR-FTIR analysis of TPU/PVC/BSO-SO nanocomposites

**Fig. 2:**
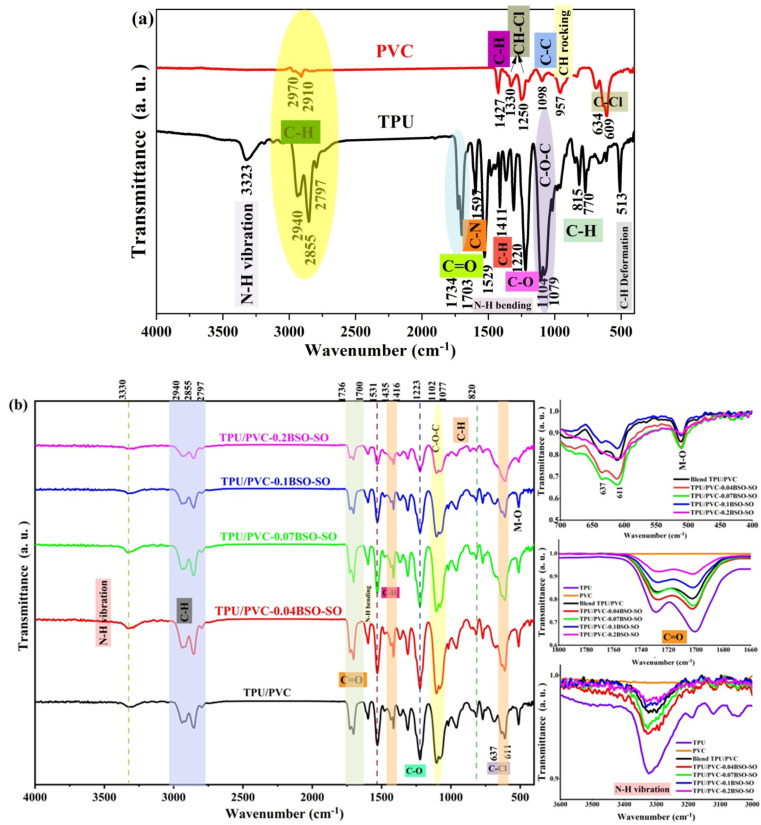
(a) The ATR-FTIR spectra of PVC and TPU. (b) The ATR-FTIR spectra of PVC/TPU blends infused with different concentrations of BSO-SO nanofiller (0.04, 0.07, 0.1, and 0.2 wt%).

Fig. 2(a) illustrates the ATR-FTIR spectra of PVC and TPU, and Fig. 2(b) shows the ATR-FTIR spectra of PVC/TPU blends infused with differing concentrations of BSO-SO nanofiller (0.04, 0.07, 0.1, and 0.2 wt%). The spectra, obtained within the region of 4000–400 cm^−1^, offer essential insights into the chemical interactions and structural modifications induced by the incorporation of BSO-SO nanoparticles.

The distinctive band of TPU is noted at 3323 cm^−1^, which relates to the stretching vibrations of the N–H group. The observed bands at 2940 cm^−1^, 2797 cm^−1^ and 2855 cm^−1^ are associated with the asymmetric and symmetric stretching vibrations of the aliphatic CH_2_ group. The bands observed between 1734 cm^−1^ and 1703 cm^−1^ are indicative of C=O bonds. The band observed at 1597 cm^−1^ signifies the in-plane stretching vibrations of C–N bonds within the aromatic rings, whereas the band at 1529 cm^−1^ is associated with N–H bending vibrations. The bands detected within the range of 1220–1253 cm^−1^ suggest the stretching vibrations of C–O bonds alongside the bending vibrations of N–H bonds. The stretching vibrations of C–O–C bonds are observed at 1104 and 1079 cm^−1^.^24–26^ PVC exhibits bands from asymmetric CH stretching at a higher frequency (∼2970 cm^−1^), while symmetric CH stretching is observed near ∼2910 cm^−1^. The bending vibrations of the C–H bond are detected at 1427 cm^−1^. Bands observed at 1330 cm^−1^ and 1250 cm^−1^ correspond to the in-plane and out-of-plane modes, respectively, of the C–H bond in CH–Cl. The C–C stretching vibrations appear at 1098 cm^−1^. The rocking vibrations of CH_2_ are seen at 957 cm^−1^. The stretching vibrations of C–Cl are observed at 634 cm^−1^ and 609 cm^−1^.^27,28^ In the TPU/PVC blends, shifts are observed in the N–H and C=O peaks of TPU, which can be attributed to its interactions with PVC. No notable new peaks emerge exclusively due to physical blending (unless chemical reactions take place), but there are very slight band shifts and noticeable changes in intensity, which indicate compatibility between TPU and PVC, as shown in Fig. 2(b). The addition of nanofiller (BSO-SO) is confirmed by a notable change in the intensities of all bands of TPU/PVC. The bands at 400–500 cm^−1^ are associated with metal–oxygen (M–O) stretching vibrations, particularly pertaining to Ba–O and Sn–O bonds.^29^ In addition, a new band appears at 612 cm^−1^ at higher BSO-SO nanoparticle concentration (0.2 wt%), which can be attributed to M–O stretching vibrations, as shown in Fig. 2(b) and listed in Table 1. The distinct emergence and enhancement of these bands in nanocomposite samples, as opposed to the pristine mixture, validate the effective incorporation of BSO/SO and delineate the characteristic lattice vibrations of the nanofiller structure.

**Table 1:** The assignment of selected ATR-FTIR peaks (4000–400 cm^−1^) from the samples

Wavenumber (cm^−1^)	Assignment	Material	Reference
∼3323	N–H stretching	TPU	24–26
2940, 2855, 2797	CH_2_ stretching (*asym*./*sym*.)	TPU	24–26
1734–1703	Urethane C=O stretching	TPU	24–26
∼1597	Aromatic C–N stretching	TPU	24–26
∼1529	N–H bending	TPU	24–26
1220–1253	C–O stretching/N–H bending	TPU	24–26
1104, 1079	C–O–C ether stretching	TPU	24–26
∼2970	CH asymmetric stretching	PVC	24–26
∼2910	CH symmetric stretching	PVC	27 and 28
∼1427	CH_2_ bending (scissoring)	PVC	27 and 28
∼1330, ∼1250	CH deformation in CH–Cl	PVC	27 and 28
∼1098	C–C stretching	PVC	27 and 28
∼957	CH_2_ rocking	PVC	27 and 28
634, 609	C–Cl stretching	PVC	27 and 28
400–500	M–O stretching (Ba–O, Sn–O)	BSO/SO	29
∼612	Additional M–O stretching at high filler loading	BSO/SO	

At lower nanoparticle concentrations (0.04 and 0.07 wt% BSO/SO), the intensities of the FTIR peaks show a slight increase. At these loadings, nanoparticles establish robust contact with the functional groups of TPU/PVC (C=O, N–H, and C–Cl), leading to the development of coordination complexes and hydrogen bonds.^30–32^ These interactions, particularly for C=O stretching and N–H and C–Cl wagging/bending/stretching, enhance the alteration of the dipole moment during vibrations.^33–35^ At elevated nanoparticle concentrations (0.1 and 0.2 wt% BSO/SO), FTIR peak intensities diminish. At higher concentrations, BSO/SO nanoparticles tend to clump together, which limits the surface area available for interaction. Additionally, most of the reactive sites on the polymer chains (–NH, C=O, and C–Cl) are already saturated.

This shows that adding BSO/SO nanofiller to TPU/PVC blends significantly affects the spectral characteristics, suggesting that there is a physical interaction at the molecular level. The patterns we found match what other studies say about polymer-metal oxide nanocomposites, showing that better bonding at the interface leads to improved composite performance.^36^

### 3.3 XRD and FESEM investigations of TPU/PVC/BSO-SO nanocomposites

**Fig. 3:**
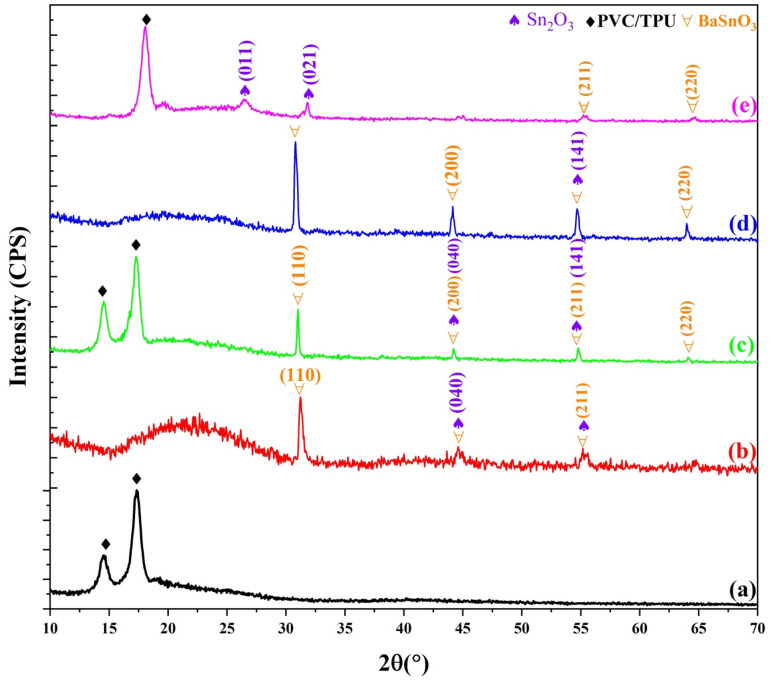
The XRD patterns of (a) the PVC/TPU blend and blends infused with different concentrations of BSO-SO nanofiller: (b) 0.04, (c) 0.07, (d) 0.1, and (e) 0.2 wt%.

The X-ray diffraction (XRD) patterns illustrate the structure of the polyvinyl chloride/thermoplastic polyurethane (PVC/TPU) composites containing varying quantities of BaSnO_3_/Sn_2_O_3_ nanocomposite. The PVC/TPU blend has a broad, indistinct peak centered between 15 and 20°, with two distinct peaks at 2*θ* values of 14.5° and 17.35°, indicating that the polymer matrix possesses partial crystallinity,^37^ as illustrated in Fig. 3(a).

With the introduction of BaSnO_3_/Sn_2_O_3_ nanoparticles at various loadings (0.04, 0.07, 0.1, and 0.2 wt% BSO/SO), separate diffraction peaks appear and increasingly intensify as the nanocomposite concentration increases. The diffraction peaks associated with BaSnO_3_ correspond to the cubic perovskite phase, as indicated by JCPDS card no. 74-130, and the triclinic phase of Sn_2_O_3_, indexed by JCPDS card no. 25-1259.^12,21–23^ The peaks are distinctly defined and become more pronounced with increasing BSO/SO content, confirming the successful incorporation and crystalline nature of BaSnO_3_ within the polymer matrix. However, at 0.2 wt%, the intensity diminishes and some peaks disappear due to the saturation of most reactive sites on the polymer chains, as corroborated by ATR-FTIR data.

At low to moderate concentrations of filler (for instance, 0.04 and 0.1 wt% BSO/SO), BaSnO_3_/Sn_2_O_3_ nanoparticles have the capacity to disrupt the semi-crystalline regions of the TPU/PVC matrix. These nanoparticles interfere with the conventional arrangement of polymer chains, especially in PVC segments that display a degree of crystallinity. This disruption results in a less-defined appearance shown by the diffraction data. At intermediate filler loadings, the presence of pronounced, distinct peaks from BaSnO_3_ and Sn_2_O_3_ may diminish the intensity of the crystalline peaks associated with TPU/PVC. At an elevated loading of 0.2 wt%, there is a propensity for nanoparticles to agglomerate, consequently diminishing the available surface area. Reduced interaction with polymers leads to the reconfiguration of chains. A degree of crystallinity is restored, leading to the reappearance of XRD peaks.

**Fig. 4:**
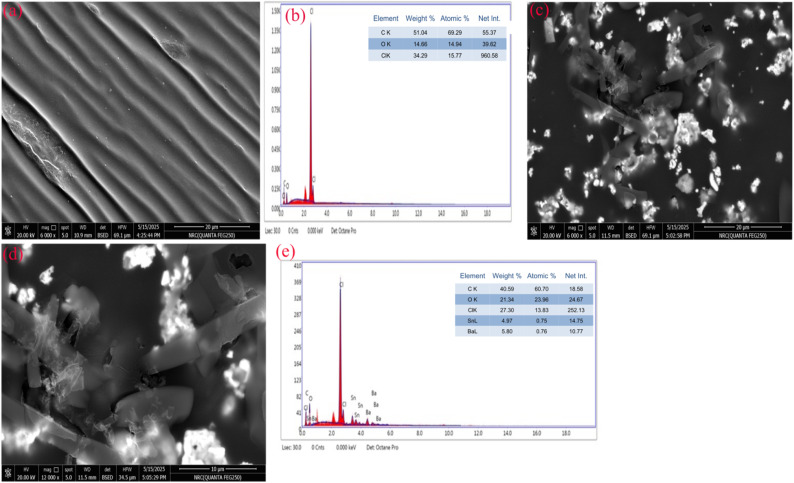
FESEM micrographs and EDX analysis of (a and b) the pristine PVC/TPU blend and (c–e) the blend infused with 0.2 wt% BSO-SO nanocomposite.

Fig. 4(a–e) presents FESEM micrographs and EDX spectra of the PVC/TPU blend before and after incorporation of 0.2 wt% BaSnO_3_-Sn_2_O_3_ (BSO-SO) nanofillers. Fig. 4(a) presents an FESEM micrograph of the pristine PVC/TPU blend at a magnification of 6000×. The micrograph displays a smooth, aligned surface morphology characterized by consistent striations, indicating high miscibility and phase compatibility between the PVC and TPU matrices. Fig. 4(b) presents the EDX spectrum of the pure blend, indicating the presence of carbon (C) and oxygen (O) as primary components, aligning with the organic polymeric characteristics of PVC and TPU. The detection of a chlorine (Cl) peak indicates the presence of PVC. Fig. 4(c) and (d) present FESEM micrographs of a PVC/TPU blend containing 0.2 wt% BSO-SO nanofiller, captured at magnifications of 6000× and 120 00×, respectively. The images illustrate notable morphological variation from the pristine blend, characterized by the presence of dispersed inorganic nanorods of nanocomposite particles and agglomerated nanofiller clusters within the matrix. This demonstrates the effective incorporation of nanofiller and indicates potential interactions between the polymer matrix and BSO-SO particles at the interface. Fig. 4(e) presents the equivalent EDX spectrum of the nanofiller-infused blend, indicating the presence of new elemental peaks from barium (Ba) and tin (Sn), alongside carbon, oxygen, and chlorine. The detection of Ba and Sn confirms the effective incorporation of BaSnO_3_ and Sn_2_O_3_ nanofiller. The distribution of these elements, along with the observed morphological changes, indicates that the nanofiller was effectively integrated. The combined FESEM and EDX analyses demonstrate the effective blending of PVC/TPU and the uniform integration of BSO-SO nanofiller, resulting in a modified microstructure that may enhance material performance.

### 3.4 Dielectric exploration

**Fig. 5:**
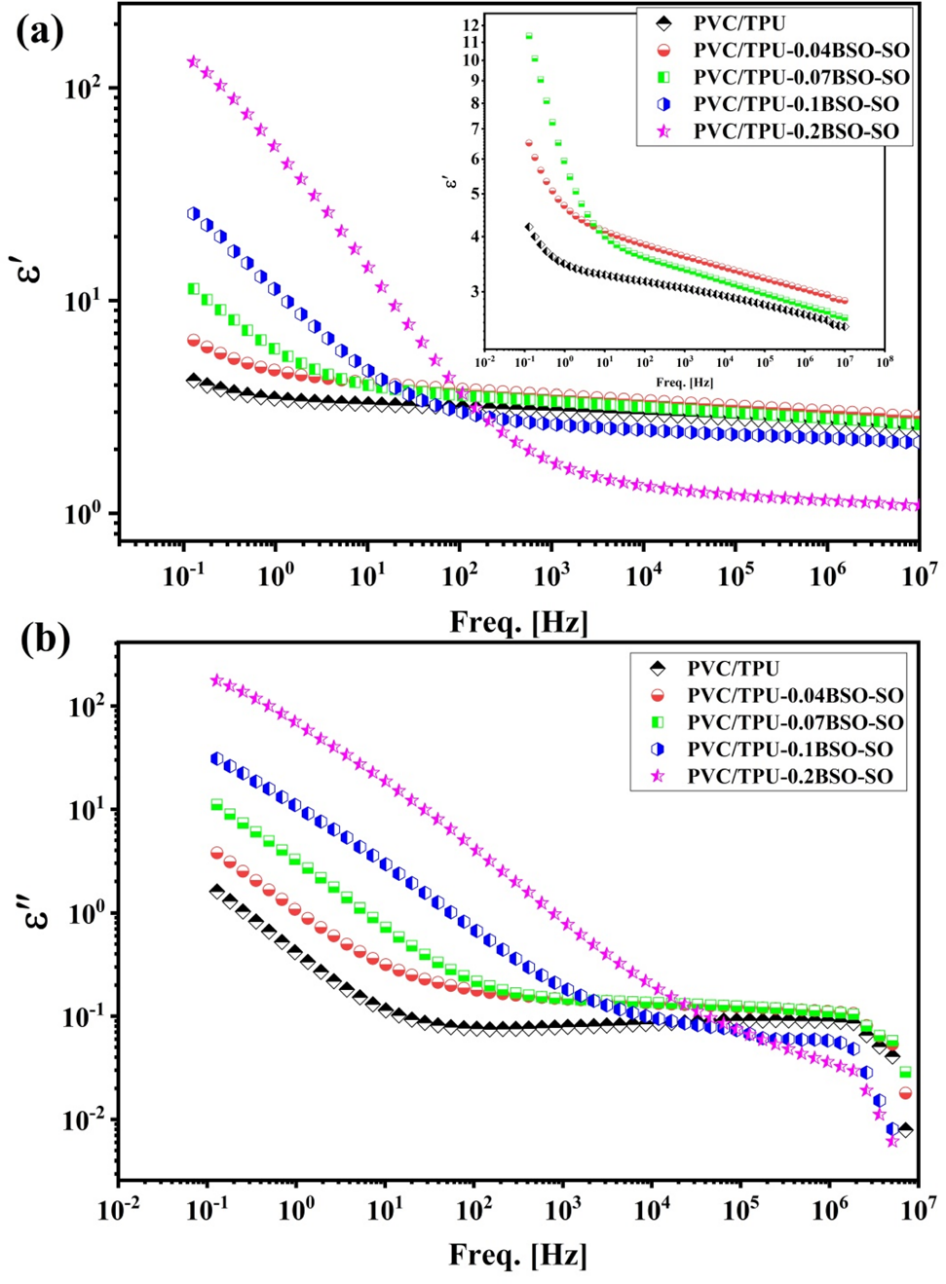
The (a) real and (b) imaginary parts of permittivity as a function of frequency at room temperature for the samples.

The dielectric characteristics of PVC/TPU blends influenced by BaSnO_3_-Sn_2_O_3_ (BSO-SO) nanofiller are studied over a broad frequency range, which, as illustrated in Fig. 5(a) and (b), spans from 10^−2^ to 10^7^ Hz at room temperature.

The energy-storage ability of the material, shown by the real part of the dielectric constant (*ε*′), decreases with increasing frequency for all samples, as shown in Fig. 5(a). The observed frequency-dependent behavior primarily stems from the diminishing effectiveness of interfacial (Maxwell–Wagner–Sillars) polarization at higher frequencies.^38,39^ This behavior is typical of dielectric materials. The plain PVC/TPU combination demonstrates the lowest *ε*′ value, approximately 3.2, at low frequencies, specifically around 10^−1^ Hz. Conversely, the sample containing 0.2 wt% BSO-SO shows a significantly higher *ε*′ value of approximately 120. This figure suggests that the dielectric permittivity of the material has been substantially enhanced by the nanofiller. The value of *ε*′ consistently increases throughout the frequency range from 10^−1^ to approximately 10^2^ Hz as the percentage of BSO-SO in the mixture increases from 0.04 to 0.2 wt%. The value of *ε*′ increases from approximately 4.5 for 0.04 BSO-SO to approximately 20 for 0.1 BSO-SO as the frequency increases to 1 Hz, and then to approximately 120 for 0.2 BSO-SO. This evidence suggests that the incorporation of nanofiller has led to enhanced polarization effects. The results we see are due to the larger surface area between the polymer matrix and the nanofiller, which helps build up space charge.^40,41^ The inset of Fig. 5(a) demonstrates the substantial separation and trend in dielectric constant at low filler concentrations.

Fig. 5(b) presents the imaginary component of the dielectric constant (*ε*″). This component illustrates dielectric loss, characterized by the dissipation of energy as heat within the material. The value of *ε*″ exhibits a decrease with increasing frequency, similar to *ε*′, suggesting that energy losses attributed to dipolar relaxation and interfacial charge movement are more pronounced at lower frequencies. The *ε*″ value peaks at approximately 90 for PVC/TPU-0.2BSO-SO at very low frequencies (around 10^−2^ Hz), whereas it drops to about 0.8 for neat PVC/TPU. This indicates that the presence of BSO-SO enhances polarization while simultaneously elevating dielectric loss. The observed phenomenon can be attributed to the formation of new conductive pathways and localized charge hopping that takes place at the interface between the polymer and the filler.^41,42^ The *ε*″ values for all samples tend to align within a lower range (approximately 0.01–0.05) as the frequency approaches 10^4^ Hz. The narrower range suggests that energy dissipation is constrained at these higher frequencies, attributable to the restricted dipolar response time.

The phenomena that have been seen have revealed that the BSO-SO nanofiller has a significant impact on the dielectric properties of PVC/TPU blends. This is demonstrated by the fact that when the concentration of nanofiller is increased, there is a corresponding general increase in both the *ε*′ and *ε*″ values. This increase can be attributed to interfacial polarization, greater dipole alignment, and increased charge carrier mobility. The conductive and high-permittivity qualities of BSO and defect-rich SO are responsible for these phenomena, which are a result of those properties. It is feasible for the system to have excessive filler loading, which suggests that it could perform better than traditional polymers in terms of its capacity to store energy.^43^

**Fig. 6:**
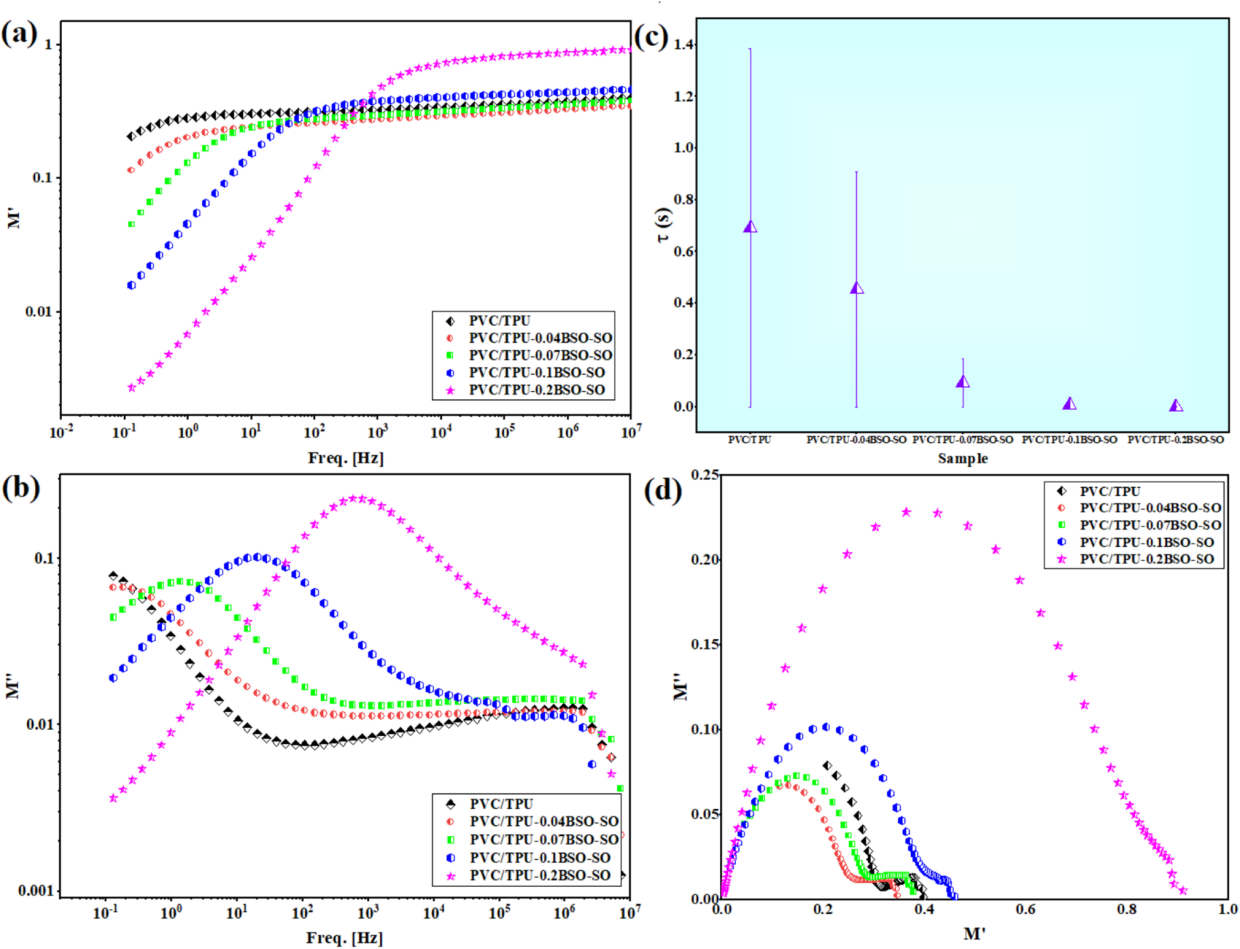
(a and b) *M*′ and *M*″ as a function of frequency, (c) the relaxation times of the samples calculated from the changes in *M*″ with frequency, and (d) Cole–Cole plots of a PVC/TPU blend and blends infused with different concentrations of BSO-SO nanofiller: 0.04, 0.07, 0.1, and 0.2 wt%.

Fig. 6(a) shows a clear pattern where the real part of the electric modulus (*M*′) goes up with frequency across all samples. In the unaltered PVC/TPU mix, *M*′ values stay low at low frequencies because of noticeable electrode polarization effects and high charge movement. *M*′ increases with the increase in frequency because the polymer chains cannot keep pace with the rapidly changing field. The sample containing 0.2 wt% BSO-SO, which has strong interfacial contact between the polymer matrix and the highly polarizable nanofiller, shows the most notable trend toward increased *M*′ values, indicating limited segmental mobility. This phenomenon suggests the development of immobilized zones around the nanofiller, therefore preventing long-range ionic conduction.^40–43^

Fig. 6(b) illustrates the imaginary component of the electric modulus (*M*″), which is responsive to relaxation phenomena within the material. The presence of a clear relaxation peak for each sample confirms that dielectric relaxation happens due to dipolar or interfacial (Maxwell–Wagner–Sillars, MWS) polarization. The peak moves to higher frequencies as the amount of BSO-SO increases, showing that the time for dielectric relaxation (*τ*) gets shorter. This trend happens because the stronger local electric fields and interfacial polarization at the boundaries between the polymer and nanofiller help the dipoles align and the charge carriers move more easily. The larger surface area and conductive features of the BSO-SO nanoparticles likely provide extra pathways for collecting and releasing charges, which accelerates the process of polarization switching.

As shown in Fig. 6(c), the relaxation periods (*τ*; *τ* = 1/2*πf*_max_) derived from the *M*″ peak positions exhibit a distinct diminishing trend as the nanofiller concentration increases.^42^ The pristine PVC/TPU mix demonstrates the longest relaxation time (*τ*) of approximately 0.7 s, but the PVC/TPU-0.2BSO-SO system shows a significantly shorter *τ* value of about 2.3 × 10^−4^ s, indicating a shift from slow dipolar relaxation to a more rapid interfacial relaxation process. This decrease in *τ* further substantiates the growing impact of nanofiller-induced heterogeneity and improved ionic mobility within the composite system.^42,43^

Fig. 6(d) illustrates Cole–Cole plots (*M*″ *vs. M*′), which visually demonstrate the dielectric relaxation mechanism. All systems exhibit semicircular arcs, indicative of non-Debye-type relaxation behavior. The dimensions and symmetry of the arcs fluctuate with the concentration of nanofiller. The arc related to PVC/TPU-0.2BSO-SO is the largest and highest, showing a wider range of relaxation times due to better interfacial polarization and more varied dielectric properties. The difference from ideal Debye behavior indicates that there are multiple overlapping ways in which the material relaxes, which may include both the movement of dipoles and MWS polarization at the interfaces between the polymer and filler.^42–45^

The incorporation of BSO-SO nanofiller notably modifies the dielectric relaxation characteristics of PVC/TPU blends *via* mechanisms that include interfacial polarization, limited polymer mobility, and improved charge carrier dynamics. The effects become more pronounced with increased filler loading, suggesting the possibility of optimizing dielectric performance through nanofiller engineering.

### 3.5 Optical analyses

**Fig. 7:**
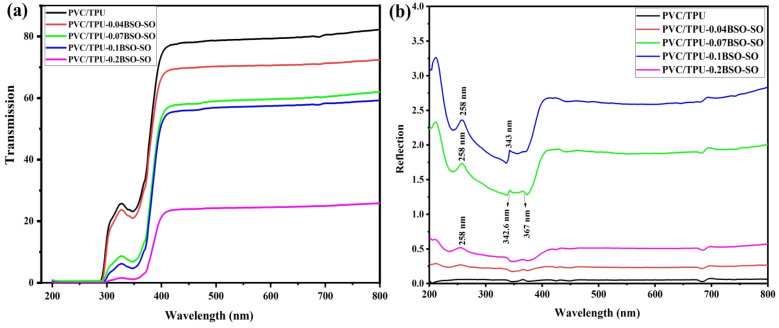
The optical transmission (a) and reflection (b) spectra of PVC/TPU polymer blends incorporating various concentrations (0.04, 0.07, 0.1, and 0.2 wt%) of BSO-SO nanocomposite.

Fig. 7 shows the transmission and reflection spectra of PVC/TPU polymer blend films with different amounts of BSO-SO (barium stannate-Sn_2_O_3_) nanofiller: 0.04 wt%, 0.07 wt%, 0.1 wt%, and 0.2 wt%. Fig. 7(a) illustrates the transmission spectra, revealing a distinct trend where the blended PVC/TPU film exhibits the highest transmission, surpassing 80% in the visible spectrum range (400–800 nm), signifying a substantial degree of transparency. As the concentration of BSO-SO nanofiller increases, the transmittance progressively diminishes. The film with 0.04 wt% BSO-SO exhibits a slight decrease in transmission, followed by more significant reductions for the 0.07 wt% and 0.1 wt% samples. The 0.2 wt% sample exhibits the most significant decline, with transmission below 30% across the visible spectrum range and under 10% in the ultraviolet region (200–400 nm). Numerous factors can explain this trend. BSO-SO nanoparticles efficiently absorb UV light due to their small bandgap and semiconductor-like behaviour, making them effective UV light blockers. The addition of high-refractive-index BSO-SO nanoparticles increases light scattering inside the film, thereby diminishing the amount of light that may pass through. Third, as more filler is added, the nanoparticles can clump together, which boosts their ability to absorb and scatter light but makes the material less transparent. This balance between absorption and scattering, especially with higher amounts of BSO-SO, provides good UV protection while reducing how much visible light can pass through, which matches what has been seen in optical films made with nanocomposites.^46^

Fig. 7(b) illustrates the UV-vis reflection spectra of unmodified PVC/TPU and nanocomposites incorporating varying concentrations of BSO-SO nanofiller (0.04, 0.07, 0.1, and 0.2 wt%). The strength of reflection fluctuates with wavelength in the range of 200–800 nm. The spectra indicate that the BSO-SO concentration influences light–matter interactions within the polymeric matrix. The PVC/TPU blend exhibited the lowest reflectance (≈0.1–0.3%) in the visible spectrum range, signifying exceptional transparency and minimal scattering. The incorporation of BSO-SO nanofiller leads to a gradual increase in reflection intensity, particularly in the UV-visible spectrum region (250–500 nm). The elevated refractive indices and optical densities of BaSnO_3_ and Sn_2_O_3_ enhance light scattering, interfacial polarization, and photon backscattering at polymer-filler interfaces. The PVC/TPU-0.1BSO-SO sample exhibits the largest reflection peak (about 3.2–3.5%), indicating effective dispersion and interaction between inorganic nanophases and organic polymer chains. The minor shoulders or humps observed at around 258 nm and in the range of 342.6 nm to 367 nm correspond to electronic transitions of Sn^4+^–O^2−^ and Ba–O charge-transfer bands, respectively, in accordance with recent optical investigations on BSO and tin oxide systems.^47,48^ These transitions arise from excitation from oxygen 2p to tin 5s/5p hybrid orbitals.^48^ The moderate reflection noted in the visible spectrum range (400–700 nm) for the 0.07 and 0.1 wt% BSO-SO samples is attributed to diverse scattering mechanisms resulting from the localized surface states and nanoscale roughness of the filler particles. Nevertheless, with an increase in filler content (0.2 wt%), the reflection intensity is somewhat diminished compared to the 0.1 wt% sample, which is possibly attributable to particle agglomeration and light trapping within clusters, which diminishes backscattering efficacy. The reflection spectra indicate that the optical response of the nanocomposites is significantly affected by filler loading, particle dispersion, and refractive index disparity between the inorganic (BSO-SO) and polymeric (PVC/TPU) phases.

**Fig. 8:**
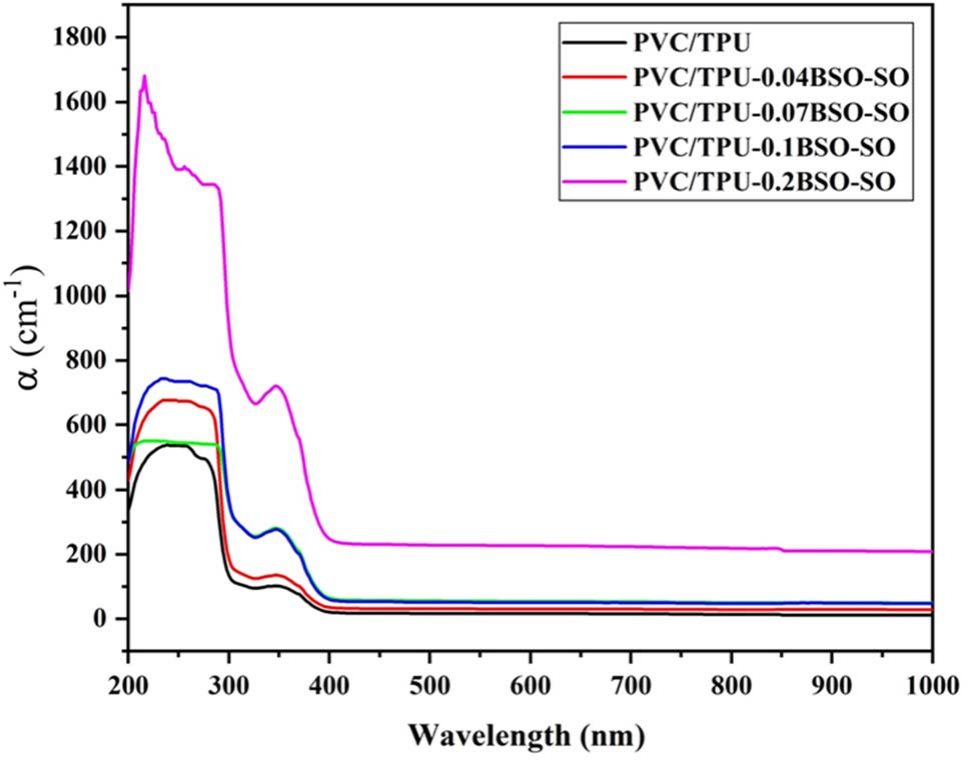
The absorption coefficient as a function of wavelength for PVC/TPU polymer blends incorporating various concentrations (0.04, 0.07, 0.1, and 0.2 wt%) of BSO-SO nanocomposite.

Fig. 8 shows how the absorption coefficient (*α*) changes with wavelength (200–1000 nm) for PVC/TPU blend films that have different amounts of BSO-SO nanofiller: 0.04, 0.07, 0.1, and 0.2 wt%. This data is obtained from transmission spectra, where the absorption coefficient, *α*,^49,50^ is calculated using the equation:1
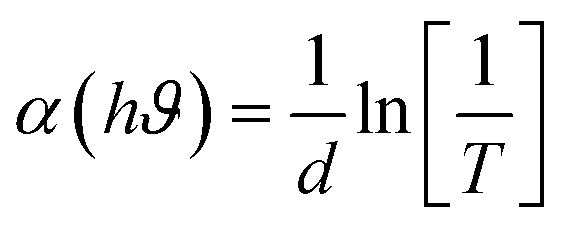
In this equation, *T* represents the percentage of transmission, while *d* denotes the film thickness measured in centimeters. The data show a big increase in *α* in the UV range (200–400 nm), indicating strong absorption and revealing an important feature called the absorption edge, related to the material's optical bandgap. The absorption edge moves slightly toward longer wavelengths (redshift) as the concentration of BSO-SO increases, which means the optical bandgap decreases, probably because of more electronic disorder and defects due to the nanofiller.

The pure PVC/TPU blend shows the least absorption in the UV range, with a clear and sharp absorption edge, indicating a well-organized system with few defects. As the amount of BSO-SO nanofiller increases, especially at 0.1 wt% and 0.2 wt%, the absorption becomes stronger, and the edge broadens. The PVC/TPU-0.2BSO-SO sample shows the highest absorption coefficient (about 1600 cm^−1^ at 250 nm), meaning there are more localized states in the bandgap area. This trend shows that adding BSO-SO nanofiller improves the film's ability to block UV light and changes its electrical structure. These materials are good for UV-blocking coatings, protective packaging, and electronic uses, like in flexible solar cells and light sensors, where it is important to have adjustable bandgaps and strong UV absorption.^46^

**Fig. 9:**
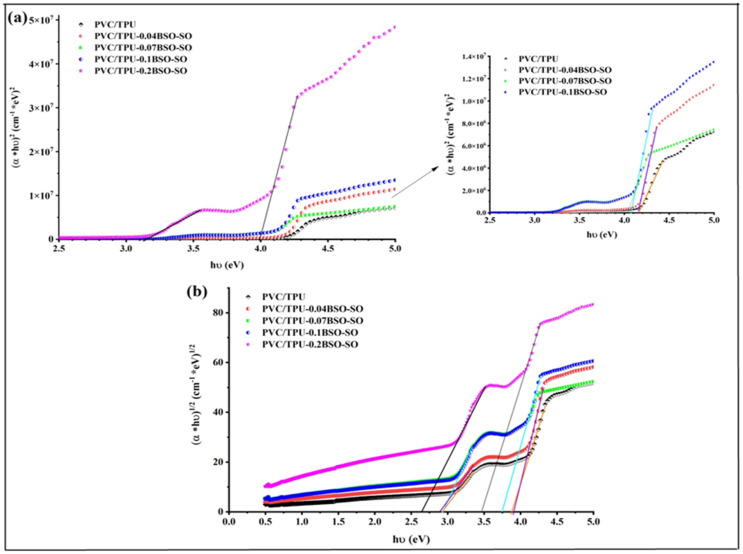
Tauc plots used to determine (a) direct and (b) indirect optical band gaps of PVC/TPU polymer blends incorporating various concentrations (0.04, 0.07, 0.1, and 0.2 wt%) of BSO-SO nanocomposite.

Fig. 9 shows Tauc plots,^49,50^ which are used to find the direct and indirect optical band gaps of PVC/TPU polymer blends mixed with different amounts of BSO-SO nanocomposite: 0.04, 0.07, 0.1, and 0.2 wt%. Fig. 9(a) and (b) show direct and indirect transitions, respectively. The unaltered PVC/TPU blend possesses a direct optical band gap of 4.17 eV, while the indirect band gap ranges from 2.9 to 3.88 eV, as shown in Table 2. The incorporation of 0.04 wt% BSO-SO results in a slight decrease in the direct band gap to 4.15 eV; however, the indirect band gap markedly increases to 2.9–3.9 eV, indicating minor alterations in the electronic structure. The addition of BSO-SO nanocomposites at concentrations of 0.07 wt% and 0.1 wt% causes significant drops in the direct band gap to 4.03 eV and 4.06 eV, respectively, while the indirect band gap decreases to between 2.75 and 3.74 eV. This suggests that adding more nanofiller improves the interactions between the polymer and nanoparticles, which may create new energy states that change how light interacts with the material. At a level of 0.2 wt% BSO-SO, the direct band gap shows two different values, changing between 3.13 and 4.00 eV, while the indirect band gap shifts to a range of 2.65 to 3.48 eV. The changes in band gaps might be due to more disorder, changes in crystal structure, and the possible clumping of nanoparticles, creating more complex energy levels. The current trend shows that adding BSO-SO nanocomposite to the PVC/TPU mixture changes the way light interacts with the material by affecting both specific and overall electronic states. The variable optical band gaps demonstrated in this study point out the advantages of these nanocomposite films for optoelectronic applications, including UV blocking, sensors, and photonic devices.^19,51,52^

**Table 2:** The direct and indirect optical band gaps calculated from Tauc plots

Sample	Direct gap (eV)	Indirect gap (eV)
PVC/TPU	4.17	2.9–3.88
PVC/TPU-0.04BSO-SO	4.15	2.9–3.9
PVC/TPU-0.07BSO-SO	4.03	2.75–3.74
PVC/TPU-0.1BSO-SO	4.06	2.75–3.74
PVC/TPU-0.2BSO-SO	3.13–4	2.65–3.48

**Fig. 10:**
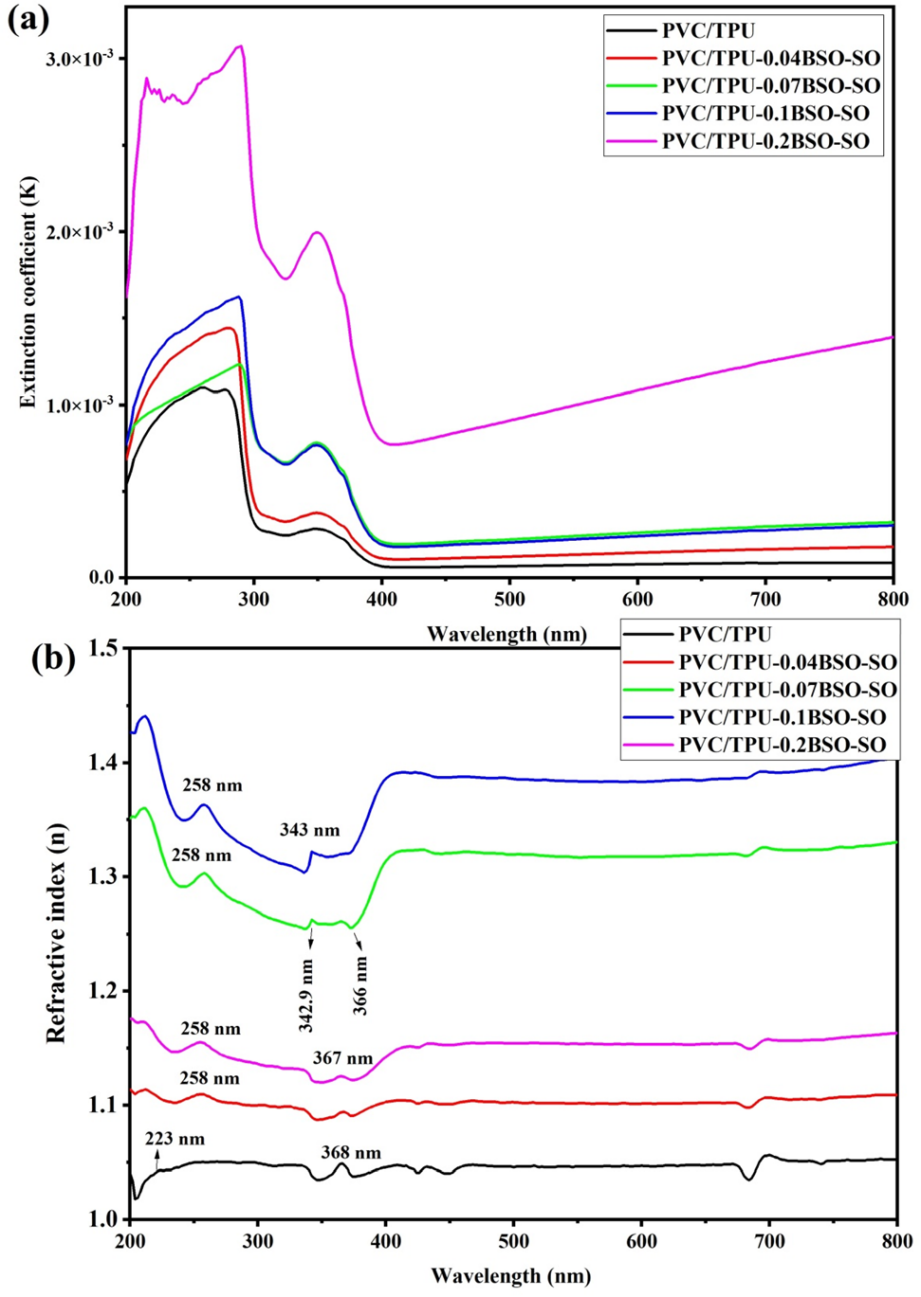
(a) The extinction coefficient (*k*) and (b) the refractive index (*n*) as functions of the wavelength for the samples.

Fig. 10 depicts the extinction coefficient (*k*) and refractive index (*n*) as functions of wavelength from 200 to 800 nm, offering profound insights into the interaction of light with these nanocomposite materials according to the following formulas:^53,54^2
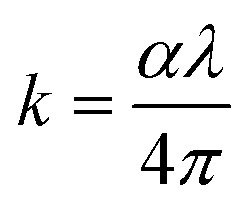
3
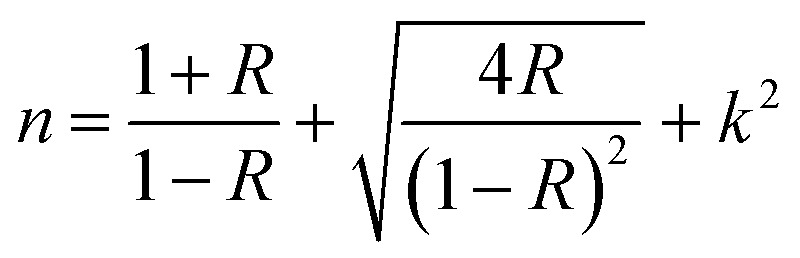


Fig. 10(a) displays the extinction coefficient, indicating the material's capacity to attenuate light through absorption and scattering. The plot indicates that all samples exhibit relatively elevated extinction coefficients in the ultraviolet (UV) range (200–400 nm) and a swift decline in the visible range (400–800 nm), characteristic of polymeric materials with broad band gaps. The pure PVC/TPU blend demonstrates the lowest *k* values, indicating negligible intrinsic optical absorption. The incorporation of BSO-SO nanofiller markedly enhances the extinction coefficient, particularly in the ultraviolet range. At 0.04 wt% BSO-SO, a slight but discernible increase in *k* is observed, suggesting improved absorption attributed to the inorganic filler, which may create localized states and interface-related transitions. With an increase in the filler concentration to 0.07 wt% and subsequently to 0.1 wt%, a consistent rise in the extinction coefficient is noted, indicating enhanced absorption in the UV range. This improvement results from enhanced dispersion and a stronger connection between the BSO-SO nanofiller and the polymer matrix, which creates additional absorption centers and may facilitate interband transitions or charge-transfer interactions. At 0.2 wt% BSO-SO, the extinction coefficient attains its maximum values, signifying a very absorptive system. Nonetheless, the curve exhibits greater irregularity and displays characteristics that may indicate the heightened dispersion or aggregation of nanofiller. This concentration-dependent trend demonstrates that the BSO-SO nanofiller markedly enhances the UV absorption capabilities, rendering these composites exceptional candidates for use in UV-shielding materials, optical filters, and light-harvesting layers in optoelectronic devices.^55^

Fig. 10(b) demonstrates the refractive index, which characterizes a material's capacity to bend light and is intimately associated with its electronic polarizability and density. All the samples exhibit normal dispersion behavior, which is characterized by a progressive decrease in the refractive index as the wavelength increases, in accordance with standard Cauchy or Sellmeier dispersion laws.^56^ Numerous spectral characteristics are discernible, each of which corresponds to distinctive electronic transitions in the polymeric and hybrid systems. The *π* → π* transition in the C=C and C=O conjugated structures of PVC and TPU chains is represented by the first band at approximately 223 nm, which is observed in pure PVC/TPU.^57^ The prominent peaks and shoulders observed at around 258 nm and between 342.6 nm and 367 nm correspond to the electronic transitions of Sn^4+^–O^2+^ and Ba–O charge-transfer bands, respectively. This aligns with recent optical investigations conducted on bismuth subcarbonate (BSO) and tin oxide systems.^46–48^ The transitions arise from excitation from oxygen 2p to tin 5s/5p hybrid orbitals.^48^ Notably, the sample containing 0.2 wt% BSO-SO exhibits a marginal reduction in refractive index compared to the 0.1 wt% sample. This may suggest that filler agglomeration occurs at elevated concentrations, leading to the formation of non-uniform domains or microvoids that reduce the overall optical density and disrupt local field homogeneity. This behavior indicates an ideal filler threshold, above which optical advantages may be undermined by structural defects or phase segregation.

The observed decrease in the refractive index to values below 1.5 shows that the PVC/TPU-BSO-SO nanocomposites have lower optical density and electronic polarizability compared to ordinary glass (*n* = 1.5–1.6). This reduction implies that light passes more freely through the materials, increasing optical transparency and reducing interfacial reflection losses, as confirmed by Tawfilas M *et al.*^58^ As a result, these nanocomposites show promise for optically transparent and anti-reflective coating applications rather than in high-index optical devices.^42–54,59^

**Fig. 11:**
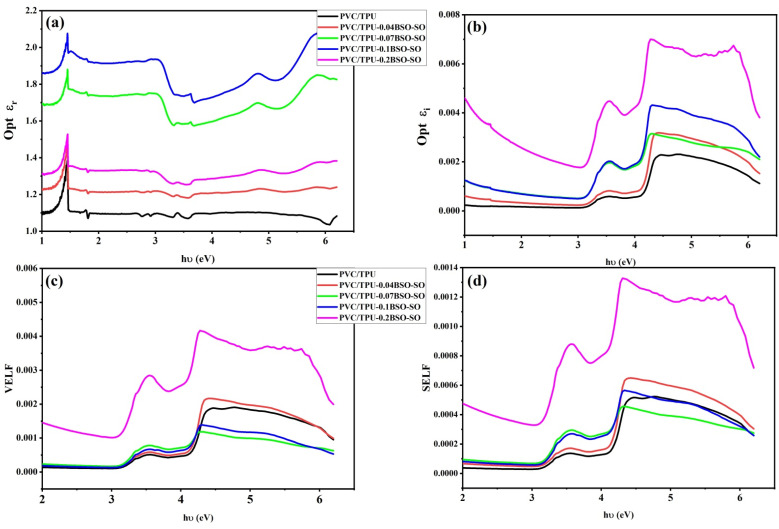
(a) The optical real dielectric constant *ε*_r_, (b) imaginary dielectric constant *ε*_i_, (c) VELF and (d) SELF as a function of photon energy for the samples.

Fig. 11 depicts the optical dielectric properties and energy dissipation characteristics of PVC/TPU polymer blends, including different concentrations (0.04, 0.07, 0.1, and 0.2 wt%) of BSO-SO nanocomposite. The real (*ε*_r_) and imaginary (*ε*_i_) components of the dielectric constant^52–54^ are obtained from the refractive index (*n*) and extinction coefficient (*k*) by the following equations:

4 *ε*_r_ = *n*^2^ − *k*^2^

5 *ε*_i_ = 2*nk*

Fig. 11(a) illustrates the real component of the dielectric constant (*ε*_r_), indicating the material's capacity to store electrical energy. As the photon energy (*hν*) increases, *ε*_r_ exhibits an initially strong increase at lower energies, followed by a plateau or mild fluctuations. The use of BSO-SO nanofiller typically enhances *ε*_r_, with the maximum values recorded for the 0.1 and 0.2 wt% composites. The improvement in *ε*_r_ is due to heightened polarizability and interfacial interactions between the polymer matrix and uniformly dispersed BSO-SO nanoparticles, resulting in enhanced dielectric performance.

Fig. 11(b) illustrates the imaginary component of the dielectric constant *ε*_i_, indicating the material's energy dissipation or absorption properties. A significant rise in *ε*_i_ is noted between 4 and 6 eV, especially for the PVC/TPU-0.2BSO-SO sample, signifying enhanced optical absorption, which is attributable to increased electronic and interband transitions prompted by the nanofiller.

The surface energy loss function (SELF) and the volume energy loss function (VELF) are shown in Fig. 11(c) and (d), respectively. The real and imaginary components of the dielectric constant are used to define the VELF and SELF, which can be expressed using the following equations:^49,50^6
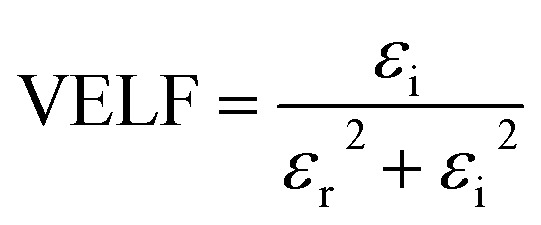
7
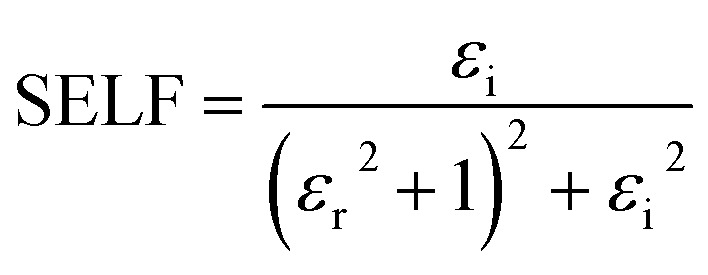


The VELF, which quantifies the energy dissipation of electrons in the material's bulk, exhibits substantial enhancement with rising BSO-SO content, especially at 0.2 wt%. This indicates that polarization caused by nanoparticles results in augmented plasmonic or collective oscillation behavior inside the bulk material. Correspondingly, the SELF, which quantifies energy dissipation at the material's surface, increases with BSO-SO loading, signifying enhanced surface plasmon resonance and interfacial contact. Both the VELF and SELF exhibit pronounced peaks from 3–6 eV, signifying regions of active plasmonic resonance influenced by the dispersion and concentration of nanofiller. The incorporation of BSO-SO nanocomposites modifies the dielectric and energy loss characteristics of PVC/TPU blends, with the 0.2 wt% formulation exhibiting the most pronounced changes. These modifications result in enhanced dielectric properties, increased optical absorption, and superior interfacial polarization, rendering the composites suitable for advanced optoelectronic and dielectric applications. VELF, which quantifies the energy dissipation of electrons in the material's bulk, exhibits substantial enhancement with rising BSO-SO content, especially at 0.2 wt%. This indicates that polarization caused by nanoparticles results in augmented plasmonic or collective oscillation behavior inside the bulk material.^50^ Correspondingly, SELF, which quantifies energy dissipation at the material's surface, increases with BSO-SO loading, signifying enhanced surface plasmon resonance and interfacial contact. Both VELF and SELF exhibit pronounced peaks at 3–6 eV, signifying regions of active plasmonic resonance influenced by the dispersion and concentration of nanofillers.

The incorporation of BSO-SO nanocomposites modifies the dielectric and energy loss characteristics of PVC/TPU blends, with the 0.2 wt% formulation exhibiting the most pronounced changes.^55^ These modifications demonstrate enhanced dielectric properties, increased optical absorption, and superior interfacial polarization, rendering the composites suitable for advanced optoelectronic and dielectric applications.^60^

## 4 Conclusions

A homogeneous hybrid system with outstanding structural integrity has been produced by effectively synthesizing PVC/TPU-BaSnO_3_/Sn_2_O_3_ (BSO/SO) nanocomposites that are lead-free through co-precipitation and drop casting. XRD, FTIR, HRTEM, and FESEM analyses confirmed the uniform incorporation of BSO/SO nanofillers and the robust interfacial interactions within the PVC/TPU matrix. The dielectric relaxation characteristics of PVC/TPU blends are significantly altered by the incorporation of BSO-SO nanofiller. This is achieved through mechanisms such as interfacial polarization, limited polymer mobility, and improved charge carrier dynamics. The dielectric constant reached 120 when the concentration of nanofiller was increased, which is twelve times higher at lower frequencies compared to the PVC/TPU matrix. The refractive indices and transmittance were effectively tuned by varying the nanofiller concentration, as evidenced by optical studies, which also disclosed a significant narrowing of the optical band gap. Additionally, the nanocomposites show potential for optically transparent and anti-reflective coating applications rather than in high-index optical devices. These results suggest that the engineered PVC/TPU-BSO/SO nanocomposites are promising options for energy storage and optoelectronic applications.

## Author contributions

Nawal K. Almaymoni, Eman A. Mwafy, and Ayman M. Mostafa: review & editing, writing – original draft, supervision. Ameenah N. Al-Ahmadi and Doaa Abdelhameed: software, methodology, investigation. Ayman A. O. Younes: formal analysis, data curation. Haitham alrajhi and Sherif S. Nafee: methodology, investigation.

## Conflicts of interest

The authors declare no conflicts of interest.

## Data Availability

The data supporting the findings of this study are available from the corresponding author upon reasonable request. All relevant experimental data, analysis methods, and modeling results used in this work can be provided to qualified researchers for the purpose of academic and non-commercial investigations.
